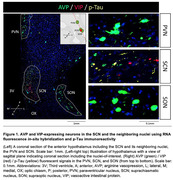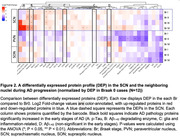# The human master circadian clock is vulnerable to tau pathology in the early stages of Alzheimer’s: a postmortem neuropathology using spatial in‐situ proteomics

**DOI:** 10.1002/alz.088431

**Published:** 2025-01-03

**Authors:** Gowoon Son, Mihovil Mladinov, Felipe Luiz Pereira, Song Hua Li, Chia‐Ling Tu, Grace Judge, Claudia Kimie Suemoto, Renata Elaine Paraizo Leite, Vitor Ribeiro Paes, Carlos Augusto Pasqualucci, Wilson Jacob‐Filho, Salvatore Spina, William W. Seeley, Wenhan Chang, Thomas C. Neylan, Lea T. Grinberg

**Affiliations:** ^1^ Memory and Aging Center, UCSF Weill Institute for Neurosciences, University of California San Francisco, San Francisco, CA USA; ^2^ San Francisco VA Medical Center, University of California San Francisco, San Francisco, CA USA; ^3^ Physiopathology in Aging Laboratory (LIM‐22), University of São Paulo Medical School, São Paulo Brazil; ^4^ Division of Geriatrics, University of São Paulo Medical School, São Paulo Brazil; ^5^ University of São Paulo Medical School, São Paulo, São Paulo Brazil; ^6^ Physiopathology in Aging Laboratory (LIM‐22), University of São Paulo Medical School, São Paulo, São Paulo Brazil; ^7^ Memory & Aging Center, Department of Neurology, University of California in San Francisco, San Francisco, CA USA

## Abstract

**Background:**

Alzheimer’s Disease(AD) patients experience circadian rhythm disorder. The circadian rhythm is synchronized by a master clock, the suprachiasmatic nucleus(SCN), which is spatially well‐conserved but a tiny nucleus in the hypothalamus. Little is known about the molecular and pathological changes that occur in the SCN during AD progression.

**Method:**

We examined postmortem brains of 12 controls without AD neuropathological changes (Braak0) and 39 subjects with progressive ADNC stages. We quantified neuronal numbers‐arginine vasopression(AVP) and vasoactive intestinal protein(VIP) neurons‐ plus ADNC burden in SCN. We performed analysis in adjacent hypothalamic nuclei as regional controls: supraoptic nucleus(SON) and paraventricular nucleus(PVN) both sites of AVP+ neurons. Moreover, we performed *In‐situ* proteomics using GeoMx Digital Spatial Profiling(DSP) in the three nuclei (total of 897 ROIs), including probes for 39 proteins commonly dysregulated in AD.

**Result:**

SCN neurons in *Braak*6 had sixteen times higher p‐tau levels than Braak0. Neurofibrillary tangles were found exclusively in SCN(Fig. 1). However, there was not significant p‐tau upregulation in SON and PVN in all the same stage. Additionally, the SCN showed increases in glial proteins already in Braak1(Fig. 2), whereas these proteins remained unaltered in the other nuclei, including that they displayed a milder pattern of protein dysregulation in Braak1, consistent with its lower tau expression observed in histology and DSP.

**Conclusion:**

Our study sheds light on the previously unexplored molecular and pathological changes occurring in SCN during AD progression. The SCN is vulnerable to AD‐tau pathology and show immune dysregulation even at Braak1 but protected against beta‐amyloid accumulation. This vulnerability pattern in SCN supports the idea that SCN dysfunction contributes to circadian rhythm disturbances in AD, observed even in the stage before the onset of cognitive disorder. Furthermore, the preservation of SON, a neighboring nucleus with AVP neurons but not directly connected in a neuronal circuit with the SCN, corroborates that SCN is early vulnerable to AD. PVN neurons exhibited similar but milder pattern of protein dysregulation in the early stages to the SCN, implying the effect may be caused by the efferent projection from the SCN. These results open up opportunities for tailored interventions to alleviate circadian rhythm disruptions in AD.